# Predictions of bitcoin prices through machine learning based frameworks

**DOI:** 10.7717/peerj-cs.413

**Published:** 2021-03-29

**Authors:** Luisanna Cocco, Roberto Tonelli, Michele Marchesi

**Affiliations:** Department of Mathematics and Computer Science, University of Cagliari, Cagliari, Italy

**Keywords:** Machine learning, Cryptocurrencies, Technical indicators, Bayesian neural network

## Abstract

The high volatility of an asset in financial markets is commonly seen as a negative factor. However short-term trades may entail high profits if traders open and close the correct positions. The high volatility of cryptocurrencies, and in particular of Bitcoin, is what made cryptocurrency trading so profitable in these last years. The main goal of this work is to compare several frameworks each other to predict the daily closing Bitcoin price, investigating those that provide the best performance, after a rigorous model selection by the so-called k-fold cross validation method. We evaluated the performance of one stage frameworks, based only on one machine learning technique, such as the Bayesian Neural Network, the Feed Forward and the Long Short Term Memory Neural Networks, and that of two stages frameworks formed by the neural networks just mentioned in cascade to Support Vector Regression. Results highlight higher performance of the two stages frameworks with respect to the correspondent one stage frameworks, but for the Bayesian Neural Network. The one stage framework based on Bayesian Neural Network has the highest performance and the order of magnitude of the mean absolute percentage error computed on the predicted price by this framework is in agreement with those reported in recent literature works.

## Introduction

Unlike the volatility of traditional market assets, the volatility of cryptocurrency markets is very high, and albeit they share the characteristics of traditional stock markets, they are highly unstable. Indeed these markets are decentralized and unregulated, and also subject to manipulation.

Nowadays many are the entrepreneurs who invest in block-chain, the well known technology underlying the most popular cryptocurrencies including Bitcoin, and we can expect that this number will grow as the Bitcoin utility increases; and many are the people who speculate on the bitcoin price.

Speculating on the Bitcoin market may offer the opportunity to obtain substantial returns, but it may also entail a very high risk. So to judge the best time to enter the market is extremely important in order to get profits and not to lose too much money.

The price of Bitcoin changes every day, just like the price of fiat currencies. However the Bitcoin price changes are on a greater scale than that of the fiat currency changes. As a result to get an idea of the future price trend can be extremely important. To date, several on-line platforms make available several technical analysis tools that allow the bitcoin speculators to identify trends and market sentiment; the number of the research papers that investigate the future bitcoin price trend is increasing.

**Figure 1:**
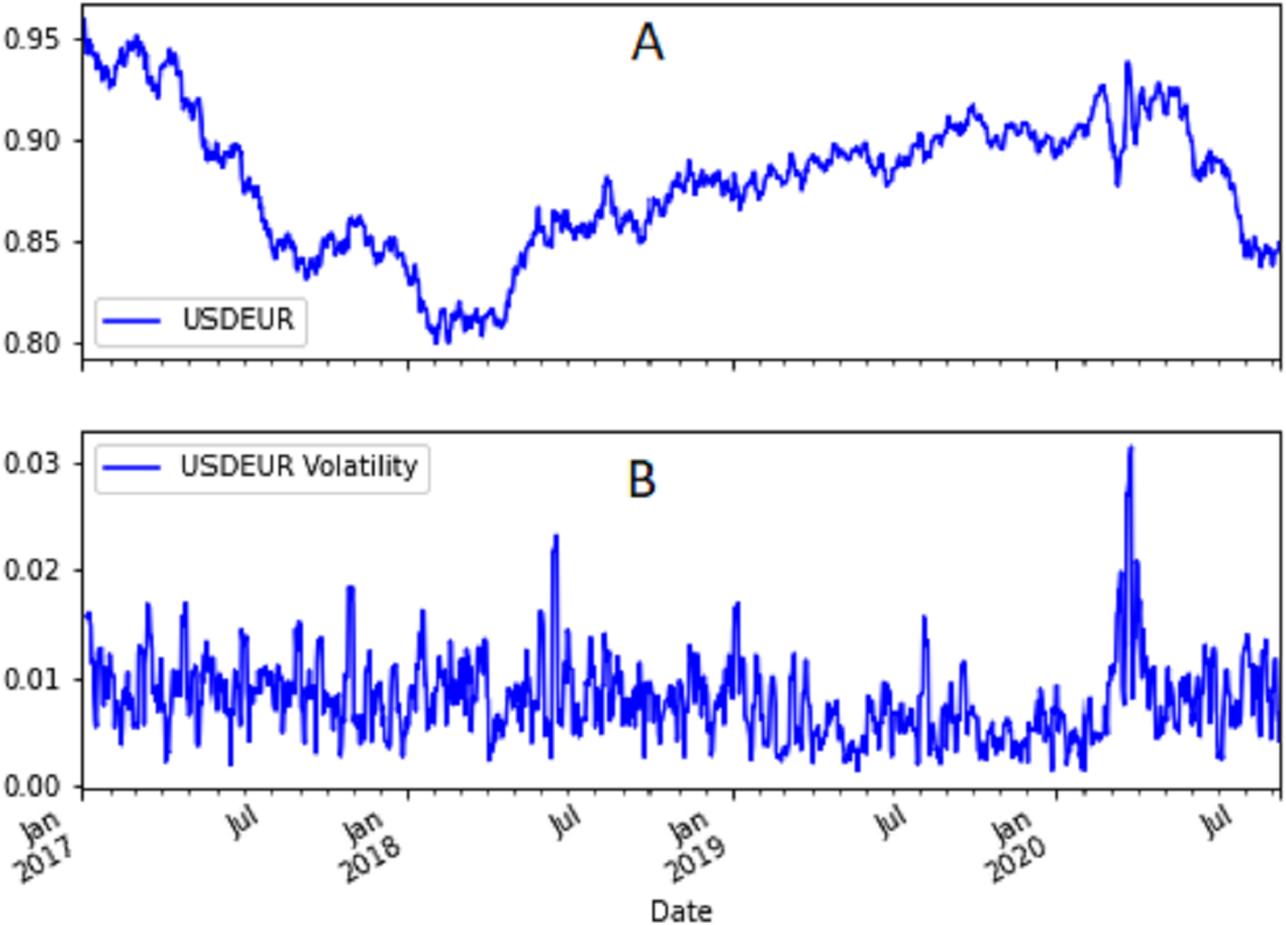
(A) Time trend of USD/EUR currency pair and (B) its volatility.

**Figure 2:**
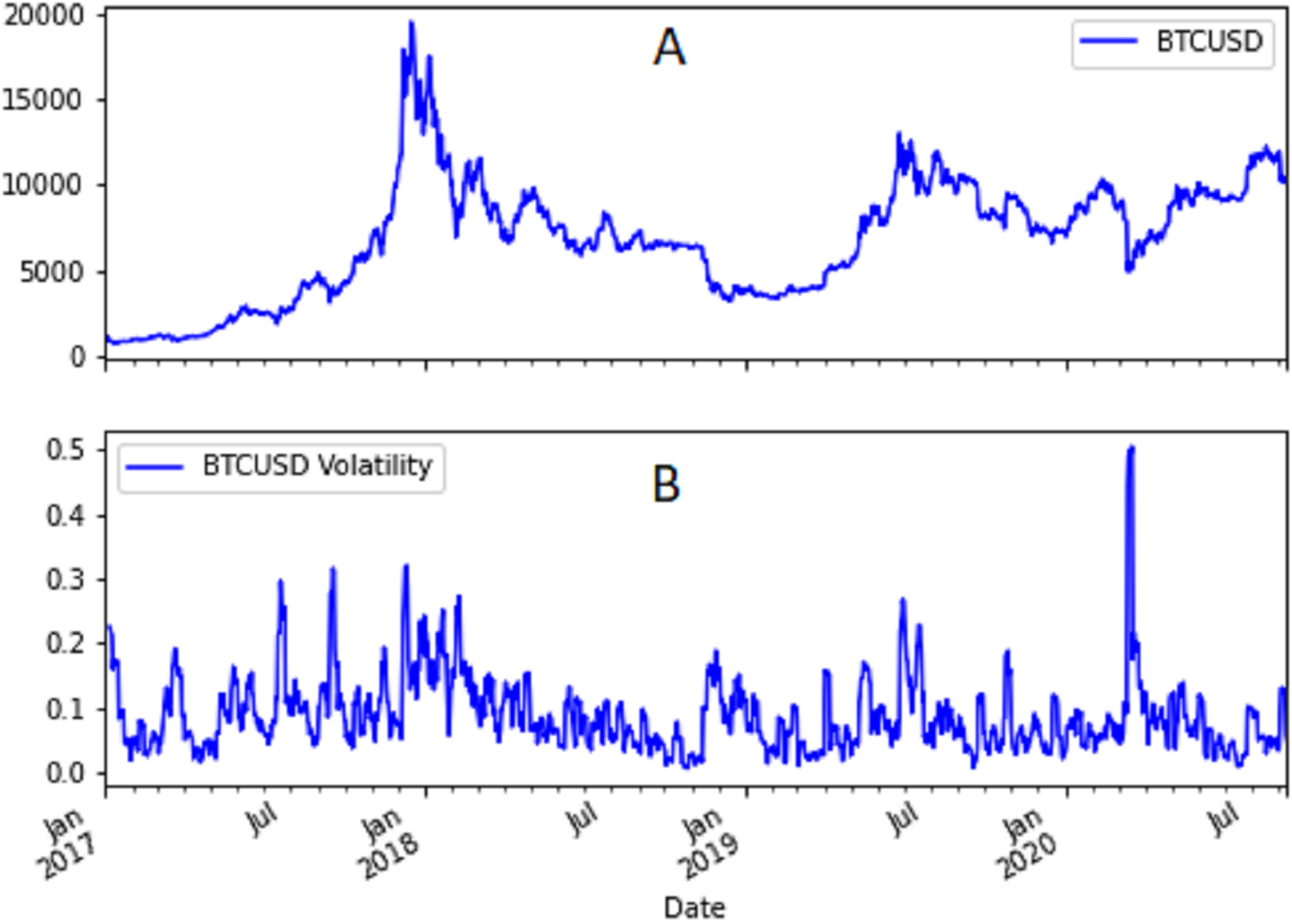
(A) Time trend of BTC/USD currency pair and (B) its volatility.

Figures 1 and 2 show the USD/EUR and BTC/USD currency pairs and their volatility. As a measure of volatility we used the moving standard deviation calculated applying the Pandas rolling standard deviation to the logarithmic returns of each just quoted currency pair using a window of 6 days. We computed also the maximum and minimum value of the USD/EUR and BTC/USD currency pairs’ volatility. The maximum value of the BTC/USD volatility is equal to 0.505, whereas the minimum value is equal to 0.005. For USD/EUR the maximum value is one order of magnitude lower. It is equal to 0.031 whereas the minimum value is equal to 0.001.

In this article we propose and study some machine learning based frameworks to forecast Bitcoin prices. These frameworks could be used to decide when and how much to invest, and also to build bitcoin trading strategies. The main goal of this work is to analyze the performance of Bayesian Neural Networks (BNN) in predicting the Bitcoin prices, and to compare them with those obtained using other kinds of NNs, such as the Feed Forward Neural Network (FFNN) and the Long Short Term Memory Neural Network (LSTMNN). In addition, following the approach proposed in the work by Patel et al. (2015), we analyzed whether the performance of the FFNN and LSTMNN increases putting each of them in cascade to another ML technique, the so called Support Vector Regression (SVR).

Let us define, as in the work by Patel et al. (2015), the first models just described, BNN, FFNN and LSTMNN, as single stage frameworks, and the others, SVR+FFNN and SVR+LSTMNN, as two stages framework. The former predicts the bitcoin price by a single ML technique, the latter instead predicts the bitcoin price by two ML techniques in cascade. All frameworks attempt to predict the Bitcoin prices starting from five technical indicators: the Simple Moving Average (SMA), the Exponential Moving Average (EMA), the Momentum (MOM), the Moving Average Convergence Divergence (MACD) and the Relative Strength Index (RSI).

**Figure 3:**
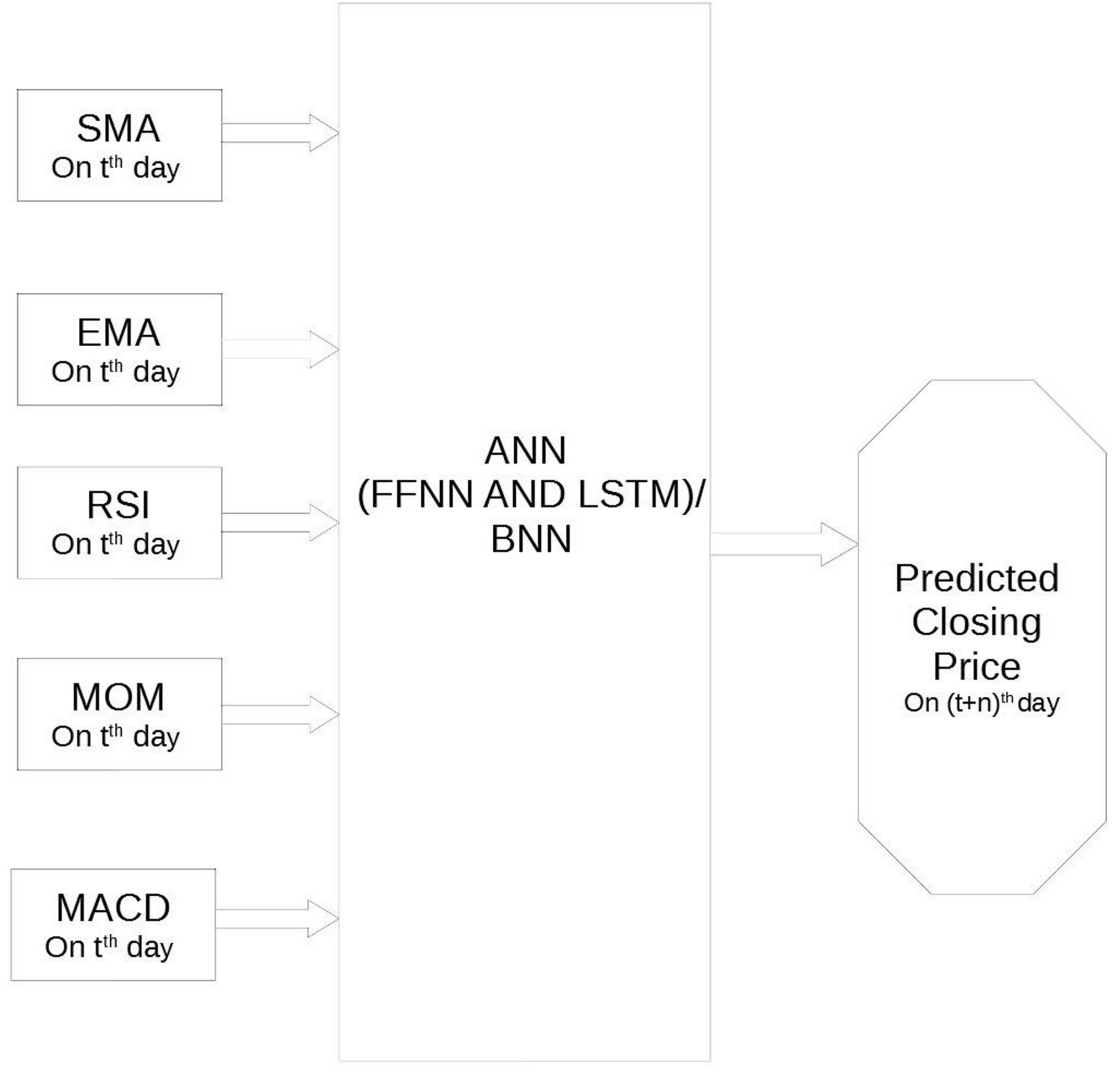
Architecture of the one stage framework.

**Figure 4:**
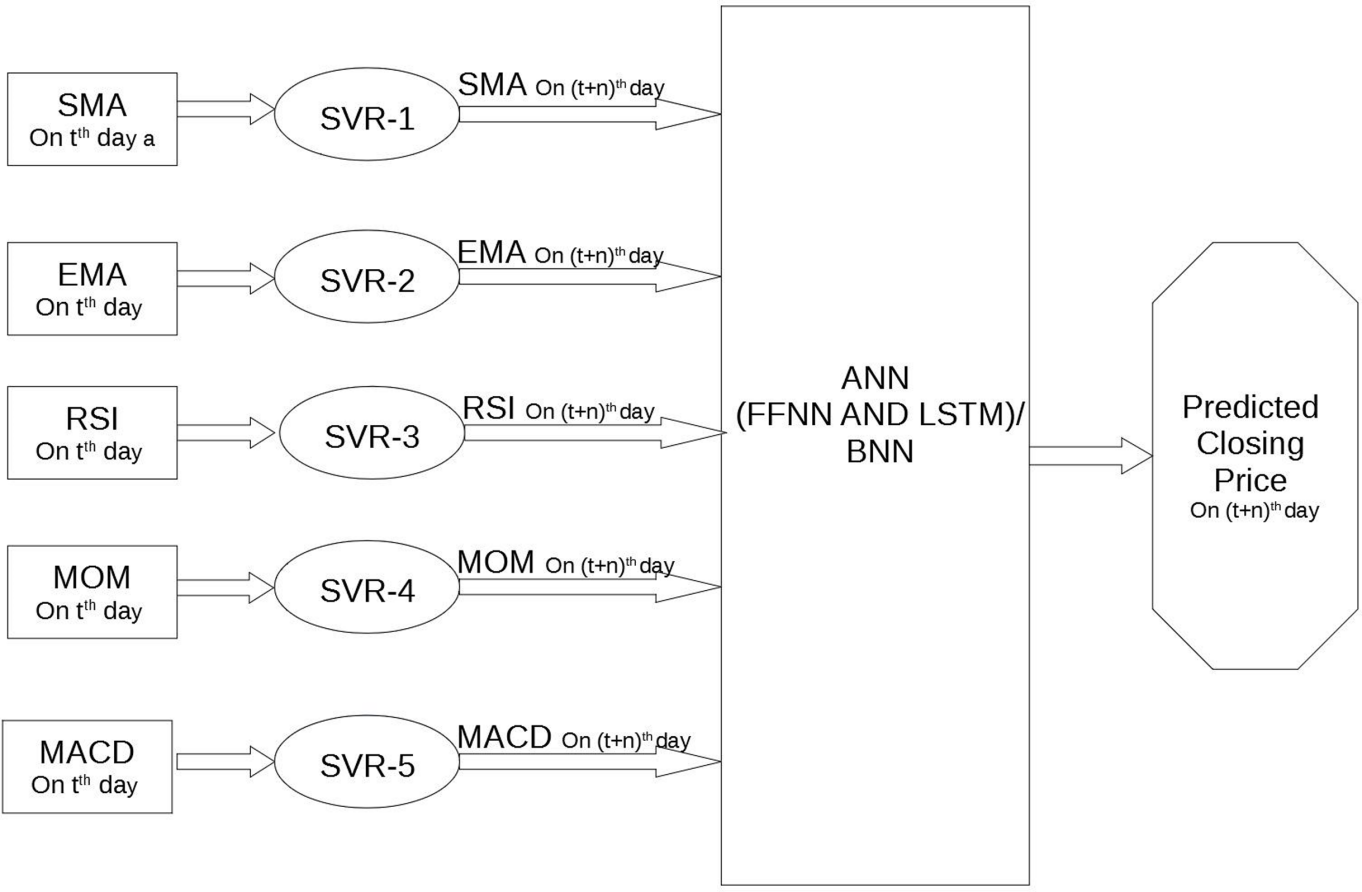
Architecture of the two stages framework.

Hence, starting from the value of these five technical indicators at *t*th day, the one-stage framework aims to predict the daily closing Bitcoin price at (*t* + *n*)th day, with *n* = 1, *n* = 10 and *n* = 20 (see Fig. 3). Instead, in the two stages frameworks the first stage, that is formed by an SVR, receives in input the five technical indicators at *t*th day and predicts the value of the five technical indicators at (*t* + *n*)th day; the second stage, that is formed by one of two NNs, receives in input the five technical indicators at (*t* + *n*)th day and predicts the daily closing price of Bitcoin at (*t* + *n*)th day^1^ 1As in all supervised learning problems, in our problem there are also input patterns (X) and output patterns (y), and given the input patterns an algorithm learns how to predict the output patterns. We transformed our time series data into a supervised learning problem using the shift() function of the well known Python data analysis library, Pandas. Starting from our time series in input, we created copies of columns of lag observations as well as columns of forecast observations, using as inputs those from *t*th days to *t* + (*n* − 1)th day and so transforming a time series dataset in a supervised learning format (see Brownlee (2017a, 2017b) for a detailed description and implementation in Python)., as in the work by Patel et al. (2015) (see Fig. 4).

To evaluate the performance of our proposed frameworks, at first we divided the whole data set into train and test set, being the test set equal to 30% of the whole data set. Then, in order to select the best neural network architectures, we calibrated these frameworks applying the *k-fold cross-validation method* to the train set just mentioned. We selected the best architecture for ANNs and the best architecture for BNNs. Once selected the best architectures, analyzing the average across *k-folds* of the mean absolute percentage error (MAPE) for each architecture, we trained the best selected architectures on all data set. Final results provide a robust estimation of the performance of these architectures, since they are the MAPE’s average (std) across the several Monte Carlo runs performed.

Let us underline that peculiarity of our work is tuning architectures’ hyper parameters by the *k-fold cross-validation method*, and predicting prices in a young, unstable and immature market such as the cryptocurrency market providing robust results thanks to the Monte Carlo method applied. Note that, we predicted the Bitcoin prices and also the Ethereum prices applying in both cases the same methodologies. In this first work, due to the computational complexity of some proposed frameworks an exhaustive optimization was not performed. Nevertheless, Bitcoin price predictions are comparable with those found in literature and proposed frameworks perform well also when applied to the Ethereum price prediction. The article is organized as follows. Related Work describes related work; the Proposed Framework section presents the frameworks proposed in our work for the prediction of bitcoin price, describing the ML techniques used and their inputs, that are the technical indicators mentioned above and that are built starting from the daily closing bitcoin price series; The Framework’s Calibration and Performance Metric section illustrates the calibration of the proposed frameworks, the training and testing data sets, and the performance metrics with which the proposed frameworks are evaluated; Results presents the results, and finally, Conclusions concludes and discusses future works.

## Related work

In this work, as already mentioned, the proposed frameworks, and in particular the idea of the approach of one and two stages, stem from the work by Patel et al. (2015). In that work, the authors predict the future values of two Indian stock market indices, CNX Nifty and S&P Bombay Stock Exchange (BSE) Sensex, by the SVR combined with Artificial Neural Network (ANN) and Random Forest (RF) algorithms. They compare the obtained results in these two stages frameworks with those obtained in the single stage frameworks formed each by a single ML technique, ANN, RF and SVR. Contrary to the work by Patel et al. (2015), in our work we investigated also the performance of the BNN.

The BNNs are taken into account in the work by Jang & Lee (2018) that use Blockchain information in order to predict the log price and the log volatility of Bitcoin price. In this work, the authors select the relevant inputs studying the multicollinearity problem, and specifically studying for each input the variance inflation factor (VIF) value. Also in a work by Mallqui & Fernandes (2019) the authors select the relevant inputs for their prediction problem, but the selection of the relevant inputs is done using the correlation analysis, the relief technique, the information gain method, the principal component analysis, and the correlation based feature subset selection. In the work by Mallqui & Fernandes (2019) the authors predict both the Bitcoin price and its movements, hence solve both a classification problem and a regression problem by different ML algorithms, Recurrent Neural Network, Tree classifier and the SVM algorithm. Owing to the low number of inputs taken into account in our work, we did not apply any selection method. We simply evaluated the performance of the proposed frameworks at varying the number of inputs taken into account, finding that the best performance of the BNN is obtained taking into account all five technical indicators. Our future work is to investigate the performance of the proposed frameworks under the assumption of a higher number of inputs, which includes also blockchain information, tweet volumes and sentiment analysis.

Twitter data and Google trends data are used by Abraham et al. (2018) in order to predict changes in the prices of both Bitcoin and Ethereum. In this work, the authors predict the direction of price changes by a linear model. In the work by Huang, Huang & Ni (2018) the authors investigated cryptocurrency return predictability, specifically the bitcoin return predictability. They forecast the bitcoin daily return using a tree-based predictive model and 128 technical indicators as input. Results showed that their predictive model has strong predictive power and performance higher than those obtained by a buy-and-hold strategy. As a result, work by Huang, Huang & Ni (2018) suggests that in the bitcoin market technical analysis can result useful.

A similar result is obtained in the work by Cocco, Tonelli & Marchesi (2019), who simulate the trading of the currency pair BTC/USD. They simulate a BTC/USD artificial market in which Chartists (speculators) trade through the application of trading rules based on technical indicators, and Random traders trade without applying any specific trading strategy. Results show that Chartists, who adopt the trading rules selected by a genetic algorithms that optimizes their parameters, are able to achieve higher profits.

Let us quote other relevant works. Shintate & Pichl (2019), Ji, Kim & Im (2019), Livieris et al. (2020), Lamothe-Fernández et al. (2020), and Chen, Li & Sun (2020) predicted Bitcoin price at different frequencies using several machine learning techniques and investigating the importance of the sample dimension. Greaves & Au (2015) investigated the predictive power of blockchain network-based features on the bitcoin price. They found a low predictive power embedded in these network features probably because Bitcoin price is technically dictated by exchanges’ behaviors. Akcora et al. (2018) introduced the concept of k-chainlets expanding the concepts of motifs and graphlets to Blockchain graphs. They found that certain types of chainlets have a high predictive power for Bitcoin prices. Lahmiri & Bekiros (2019) implemented deep learning techniques to forecast the price of the three cryptocurrencies, Bitcoin, Digital Cash and Ripple, finding that these three digital currencies exhibit fractal dynamics, long memory and self-similarity. Indera et al. (2018) presented a Multi-Layer Perceptron-based Non-Linear Autoregressive with Exogeneous Inputs (NARX) model to predict Bitcoin price starting from the opening, closing, minimum, maximum past prices and a technical indicator, the well-known Moving Average. Munim, Shakil & Alon (2019) forecasted Bitcoin price using the autoregressive integrated moving average (ARIMA) and neural network autoregression (NNAR) models. Uras et al. (2020) segmented each analyzed financial time series into short partially overlapping sequences in such a way that these sequences do not resemble a random walk. The authors identified a train and a test set within each time regime/sequence. Then, in each identified sequence they applied different forecasting procedures—Simple Linear Regression, Multiple Linear Regression, Multilayer Perceptron and the Long short-term memory. Mudassir et al. (2020) presented high-performance machine learning-based classification and regression models for predicting Bitcoin price movements and prices in short and medium terms. Among the machine learning techniques used by the authors there is the stacked ANN (SANN), constituted of 5 ANN models that are used to train a larger ANN. The SANN was trained using the training dataset and the 5-fold cross-validation, by training each of 5 ANN models on a separate fold. The final larger ANN learns from these five models, that is, it trains on the outputs of the five individual smaller ANNs. All the cited works focus on end-of-day closing price forecast and/or price movements forecasting for the next day prices, but the work by Patel et al. (2015) and the last work quoted, that by Mudassir et al. (2020). The former focuses on forecasts for 1–10, 15 and 30 days in advance, instead the latter focuses on end-of day, short-term (7 days) and mid-term (30 and 90 days) forecasts. In this work we focus on end-of day, short-term (10 days) and mid-term (20 days) forecasts.

Finally, we quote the works by Jang et al. (2018), Chih-Hung, Yu-Feng & Chih-Hung (2018), Pant et al. (2018), McNally, Roche & Caton (2016), Phaladisailoed & Numnonda (2018), Roy, Nanjiba & Chakrabarty (2018), and by Velankar, Valecha & Maji (2018) that are published in the proceedings of recent international conferences and deal with the prediction of the bitcoin price using machine learning techniques.

To the best of our knowledge, our work is the first attempt of predicting the bitcoin price investigating the best architecture by the so called *k-fold cross-validation method*, applying it only to a part of the whole dataset, as described in details in Section *Framework’s Calibration and Performance Metric*. In modern applied machine learning in order to tune model hyper parameters the definition of the *k-fold cross-validation method* often replaces the definitions of training, validation and test data set for preventing overfitting.

In their book Kuhn & Johnson (2013) wrote:

…*we must use the existing data to identify settings for the model’s parameters that yield the best and most realistic predictive performance (known as model tuning).Traditionally, this has been achieved by splitting the existing data into training and test sets. The training set is used to build and tune the model and the test set is used to estimate the model’s predictive performance. Modern approaches to model building split the data into multiple training and testing sets, which have been shown to often find more optimal tuning parameters and give a more accurate representation of the model’s predictive performance*.

This is because using a sole test set or a validation set has many limitations, as discussed by Kuhn & Johnson (2013), Brownlee (2017c), Anguita et al. (2012), and Rodriguez, Perez & Lozano (2010).

It is worth underlining that every machine learning model always has some error because it is a model. Kuhn & Johnson (2013) wrote:

*Many modern classification and regression models are highly adaptable; they are capable of modeling complex relationships. However, they can very easily overemphasize patterns that are not reproducible. Without a methodological approach to evaluating models, the modeler will not know about the problem until the next set of samples are predicted*.

In statistics a common aphorism, that is generally attributed to the statistician George Box, goes *All models are wrong* and is often expanded as *All models are wrong but some are useful*. This aphorism applies also to the choice and preparation of data, choice of hyperparameters, and the interpretation of model predictions.

In this work we attempted of predicting the bitcoin price investigating the best architecture by the *k-fold cross-validation method*, and using the Monte Carlo method to handle the stochastic nature of the neural networks, hence providing statistics to summarize the performance of the best selected models. This approach is not always applicable due to the long training times of some models. The Monte Carlo method as well as all methods and tools from probability provide a way to handle the random nature of the predictive modeling problems.

Note that, as we are going to describe in next sections, our results are comparable with those in literature despite the proposed frameworks are relatively simple in comparison to those proposed previously in literature. There are works that propose a framework using a high number of inputs, deep neural networks (DNN), convolutional neural networks (CNN) and complex stacking ensemble models (see for example the ensemble model proposed by Ji, Kim & Im (2019), in which the first level consists DNN, LSTM, and CNN, and the second level consists of a single DNN model).

## Proposed frameworks

In this work we compare the performance of the single stage frameworks, formed by an NN (BNN, FFNN, or LSTMNN), with the performance of the two stages frameworks, formed by an NN in cascade to an SVR. All frameworks aim to predict the daily closing Bitcoin and Ethereum price at (*t* + *n*)th day, with *n* = 1, *n* = 10, and *n* = 20, starting from the value of five technical indicators, SMA, EMA, MOM, RSI and MACD, at *t*th day.

In the following we briefly describe the technical indicators and the ML techniques adopted in this work.

### Technical indicators

The five indicators above mentioned are indicators well known in the technical analysis^2^ 2Technical analysis forecasts the movements of the financial assets’ prices through the study of past market data, such as price and volume. (Kirkpatrick & Dahlquist, 2006). They are mathematical constructions used to process data, for example on the price trend and on the volumes traded of a financial security in order to predict its future price performance and get buy and sell signals^3^ 3For more details see http://mrjbq7.github.io/ta-lib/funcs.html, a link in which the functions of the library *TA-Lib*, used to compute these technical indicators, are explained..

#### Moving averages

Moving averages, SMA and EMA, are basic indicators for determining price trends. They are defined as follows:

(1) }{}{\rm SMA}_t = \displaystyle{1 \over n}\sum\limits_1^n {P_t}

and

(2) }{}{\rm EMA}_t = {\rm EMA}_{t - 1} + \displaystyle{2 \over {n + 1}}({P_t} - {\rm EMA}_{t - 1})

where *P* is the price and *n* is the used period. Both are calculated by averaging a certain number of past data. The main difference is that in the EMA the data are weighted, and old data have less weight than recent data^4^ 4EMA value at *t* is based on the previous EMA value at *t* :amp:minus; 1. The initial value for EMA at *t* :amp:minus; 1 will be an SMA value calculated using a period equal to that used in EMA.. Owing to the high volatility of the bitcoin price, we dealt with short term moving averages, which usually take into account periods between 5 and 20 days. In this work we considered moving average on periods equal to 5 days.

#### Oscillators

Contrary to the moving averages, MACD, RSI and MOM are called oscillators because their value oscillates in a sinusoidal manner between a minimum and a maximum. They can be useful for identifying points of excessive price increase or excessive price decrease, and points of possible change in the direction of prices.

MACD considers the difference between the values of two moving averages (MACD line), one is a short period EMA and the other is a long period EMA, together with an exponential moving average of this difference (signal line) and a histogram given by the distance between the MACD line and the signal line. It is defined as follows:

(3) }{}shortEma = 0.15{P_t} + 0.85shortEm{a_{t - 1}}

(4) }{}longEma = 0.075{P_t} + 0.925longEm{a_{t - 1}}

and

(5) }{}{\rm MACD} = shortEma - longEma

12 and 26-day averages usually determine the MACD line, while the 9-day average determines the signal line. In this work we considered these values to compute the MACD.

RSI is a technical indicator of momentum useful for identifying the phases of oversold and overbought asset. It is defined as follows:

(6) }{}{\rm RSI} = 100\displaystyle{{upavg} \over {upavg + dnavg}}

where

}{}upavg = \displaystyle{{upav{g_{t - 1}}*(n - 1) + up} \over n}

}{}dnavg = \displaystyle{{dnav{g_{t - 1}}*(n - 1) + dn} \over n}

and *n* is the used period to compute this indicator, *up* = *P*_*t*_ − *P*_*t*−1_ and *dn* = 0 if *P*_*t*_ > *P*_*t*−1_, otherwise *up* = 0 and *dn* = *P*_*t*−1_ − *P*_*t*_. Areas of overbought indicate a time when prices go too far above their average period, and given that the price is now too high we can expect an imminent return of prices downwards (sell signal). Areas of oversold indicate a time when prices have pushed too low compared to their average and therefore we expect an imminent bullish return movement towards their average (buy signal). RSI values range from 0 to 100. Over 70 points there is an overbought signal, and under 30 an oversold signal.

MOM measures the rate of change of any instrument. It compares the current price with the price of some previous periods:

(7) }{}{\rm MOM} = {P_t} - {P_{t - n}}

In this work we set the period used by this oscillator equal to 9 days.

### Machine learning techniques

All ML techniques adopted in this work operate in a supervised context. The training takes place by presenting to the network inputs (training dataset) whose output is known, hence by presenting to the network the data set (**x**_**n**_, *y*_*n*_), where each data point in input }{}{{\bf x}_{\bf n}} \in {{\rm {\open R}}^{\rm {\open D}}}, whereas the output }{}{y_n} \in {\rm {\open R}}.

#### Bayesian neural network

Contrary to other types of neural networks, such as the FF and the LSTM, which find a weighted array that maximizes the fitting of the NN outputs with respect to training outputs, following the principle of Maximum Likelihood, the BNNs follow the Bayesian approach.

In the Maximum Likelihood, the learning aims at finding a single network—“the best”—the one that makes the smallest error on training data. As a result, at the end of the training, we get a single weight array. On the contrary the Bayesian Approach considers a probability distribution for the weights. At the end of the training we have more than one network and the output is computed as the expected value of the outputs generated by all these networks.

In deeper detail, in an BNN the weights and the outputs are assumed to be sampled from a distribution. Let us define the BNN taken into account in this work. It has an input with }{}{\rm {\open D}} dimensions and two hidden layers, whose dimensions are *HL*_1_ and *HL*_2_, respectively. For such a network the parameters }{}{\rm \theta} = \left\{ {{{\rm \omega} _{\bf 0}},{{\rm \omega} _{\bf 1}},{{\rm \omega} _{\bf 2}},{{\bf b}_{\bf 0}},{{\bf b}_{\bf 1}},{\bf b_2}} \right\} are defined as follows:

}{}{{\rm \omega} _0} \in {{\rm {\open R}}^{{\rm {\open D}} \times H{L_1}}},{{\rm \omega} _1} \in {{\rm {\open R}}^{H{L_1} \times H{L_2}}},{{\rm \omega} _2} \in {{\rm {\open R}}^{H{L_2} \times 1}},{b_0} \in {{\rm {\open R}}^{H{L_1}}},{b_1} \in {{\rm {\open R}}^{H{L_2}}},\ {\rm and}\ {b_2} \in {\rm {\open R}}

The activation functions that allow to pass from a layer to another are defined by rectified linear unit activations, hence our NN network is defined as follows^5^ 5See http://edwardlib.org/tutorials/bayesian-neural-network and https://github.com/mikkokemppainen/Jupyter_notebooks/blob/master/Edward_notebook_public.ipynb.:

}{}NN:{{\rm {\open R}}^{\rm {\open D}}} \Rightarrow {{\rm {\open R}}^{H{L_1}}} \Rightarrow {{\rm {\open R}}^{H{L_2}}} \Rightarrow {\rm {\open R}}

and a data point in input **x** pass through the following layers:

}{}{\bf x} \Rightarrow {h_1} = max(0,{{\rm \omega} _{\bf 0}}^T{\bf x} + {{\bf b}_{\bf 0}}) \Rightarrow {h_2} = max(0,{{\rm \omega} _{\bf 1}}^T{\bf x} + {{\bf b}_{\bf 1}}) \Rightarrow {{\rm \omega} _{\bf 2}}^T{h_2} + {b_2}

The prior distribution on the weights is defined as a standard normal distribution, as follows:

}{}p({\rm \theta} ) = Normal({\rm \theta} |0,I)

(where 0 is a zero matrix and *I* is a identity matrix), and the likelihood for each data point (**x**, *y*) is given by:

}{}p(y|{\bf x},{\rm \theta} ) = p({\rm {\open D}}|{\rm \theta} ) = Normal(y|NN({\bf x};{{\rm \theta} _i}),{{\rm \sigma} ^2})

where }{}{\rm {\open D}} denotes the training dataset, hence it represents all data points to be presented to the network during the training, and }{}{\rm \sigma} _y^2 is a fixed variance set equal to 1.

Given the prior and the likelihood, the posterior distribution *p*(θ, **x**) is approximated by a parametrized family *q*(θ, λ) through a variational inference method^6^ 6See http://edwardlib.org/tutorials/klqp. minimizing the Kullback–Leibler divergence between the two distributions *q* and *p*. Note that the data are scaled to be centered and have unit variance.

Once estimated the posterior distribution, that is the distribution of the weights *θ* of the BNN, we can compute the posterior predictive distribution for testing data points in input **x**_**test**_. This distribution is given by:

}{}p(y|{{\bf x}_{{\bf test}}},{\rm \theta} ) = \int Normal(y|NN({\bf x};{\rm \theta} ),{{\rm \sigma} ^2})p({\rm \theta} |{\rm {\open D}})d{\rm \theta}

it can be computed using the Monte Carlo approximation:

}{}p(y|{{\bf x}_{{\bf test}}},{\rm \theta} ){\rm \sim }\displaystyle{1 \over {{n_{\rm samples}}}}\sum\limits_{i = 0}^{{n_{\rm samples}} - 1} N(y|NN({{\bf x}_{{\bf test}}},{{\rm \theta}} _i}),{{\rm \sigma}} ^2})

we sampled it from the posterior, that is from the distribution *q*(θ, λ *). This implies sampling the weights θ, which gives *NN*(**x**_**test**_, θ_*i*_) with }{}i = 0 \ldots M where *M* is the number of extracted samples.

*M* values of *NN*(**x**_**test**_,θ_*i*_), that is *M* values of *y*, are associated to each data point **x**_**test**_ belonging to the testing dataset. Since to each **x**_**test**_ in input must correspond in output only one value of *y*, we have to compute the posterior predictive distribution for each **x**_**test**_ in the testing dataset.

Due to the computational complexity, we grouped all the sample predictions into histograms forming the posterior predictive distribution as a mixture distribution according to these histograms^7^ 7A mixture distribution is the probability distribution of a random variable that is derived from a collection of other random variables as follows: first, a random variable is selected by chance from the collection according to given probabilities of selection, and then the value of the desired random variable is obtained (http://edwardlib.org/api/ed/models/Mixture).. We computed the prices’ prediction sampling from this mixture, computing the mean.

#### Feed forward and long short term memory neural network

As regards the other two neural networks taken into account in this work, the FFNN and the LSTMNN, the main difference between them is that the former is composed of a series of layers of neurons connected without cycles, whereas the latter is characterized by the presence of cycles and is able to consider long-term dependencies among data.

Specifically, we implemented an FFNN, composed of 3 layers, an input layer, a hidden layer and an output layer as in work by Patel et al. (2015). The input layer takes in input the five technical indicators, and the output layer predicts the price at (*t* + *n*)th day. We initialized the network’s weights to a random number generated from a uniform distribution, used a tangent sigmoid as activation function in the first two layers, and a linear activation function in the output layer, as in the work Patel et al. (2015). The output layer of the neural network has only one neuron and the value, it returns, is compared with the true value. We implemented an LSTMNN having a visible layer with in input the five technical indicators, a hidden layer with *n*_*n*_ neurons, also called LSTM blocks, and an output layer that predicts the price at (*t* + *n*)th day. The default sigmoid activation function is used for the LSTM blocks.

We trained both the networks for *n*_*e*_ epochs and used a batch size of *n*_*b*_ and Adam as optimization algorithm to update network weights (Kingma & Adam, 2015). This algorithm is based on the gradient descent that minimizes an objective function, in our case the mean absolute error (MAE) of the output produced by the NNs with respect to the desired output.

#### Support vector regression

Let us conclude this brief overview with the ML technique used in the two stages frameworks, SVR. It belongs to a set of supervised learning methods that can be used both for classification and for regression computation. In a problem of classification, this technique is called Support vector machines (SVM). For instance, it can be used to divide the set of training data in two classes; an SVM is able to identify the hyperplane having the maximum margin of separation between the two classes. To this purpose, the training data set is mapped in a space called *feature space* using non-linear functions, ψ, called *feature functions*, which are a combination of the so called *kernel functions*. They map a lower dimensional data into a higher dimensional data.

In a regression problem, SVR tries to keep the fitting error within a certain threshold. Indeed, the goal of this ML technique is to find a function ψ that deviates from the observed value by a value no greater than ε for each training point.

The kernel functions more used, and adopted also in this work are the following.

- *Linear*: }{}K({x_i},{y_j}) = x_i^T{y_j}.
- *Polynomial*: }{}K({x_i},{y_j}) = (\gamma x_i^T{y_j} + r{)^d}, being *d* the degree of the polynomial function.
- *Radial basis function*: *K*(*x*_*i*_,*y*_*j*_) = *exp*(−γ||*x*_*i*_ − *x*_*j*_||^2^), with γ > 0.

## Frameworks’ calibration and performance metric

We analyzed the time series of Bitcoin and Ethereum daily closing prices. Specifically, the dataset taken into account includes daily closing price’s values from January 1st, 2017 to April 30th, 2020, for a total of 1,216 values.

Starting from these series we computed the time series of the five technical indicators, that are the inputs of our frameworks, and the features **x**_**n**_ of the dataset (**x**_**n**_, *y*_*n*_), including the training and testing datasets. The bitcoin daily price time series defines the output *y*_*n*_ of such dataset. Summary statistics for the five inputs and the output are described in Table 1 for Bitcoin and Ethereum price time series.

**Table 1:** Summary statistics for the five inputs and the output for Bitcoin and Ethereum prices.

	Max	Min	Mean	Standard deviation
BTC				
SMA	18,380.97	970.58	6,584.88	3,278.79
EMA	18,166.92	977.8	6,585.17	3,270.38
MOM	5,725.74	−4,786.35	52	1,102.21
MACD	2,546.46	−1,447.3	36.69	432.44
RSI	97.84	5.91	53.72	18.66
CLOSE	19,237.15	924.1	6,597.43	3,285.75
ETH				
SMA	1,296.65	10.79	292.26	233.62
EMA	1,302.17	10.84	292.27	233.08
MOM	543.01	−452.89	1.41	76.07
MACD	173.3	−94.25	0.95	29.81
RSI	94.54	10.77	52.08	17.61
CLOSE	1,396.42	11.03	292.59	234.34

To evaluate the performance of the proposed single stage and of the two stages frameworks, we computed the Mean Absolute Percentage Error (MAPE), defined as follows:

- \displaystyle{1 \over n}\sum\nolimits_{t = 1}^n \displaystyle{{|{A_t} - {F_t}|} \over {|{A_t}|}}*100

where *A*_*t*_ and *F*_*t*_ are the actual and forecast prices, respectively, at time *t*.

As regards the calibration of the used ML techniques, we tuned the model hyper parameters, that is the parameters whose values are set before starting the learning process, using the *k-fold cross-validation method* (see works by Kuhn & Johnson (2013), Brownlee (2017c), and Rodriguez, Perez & Lozano (2010)). The *k-fold cross-validation method* implies the creation of several neural network architectures and the training of every architecture at each k-fold. For each architecture and for each k-fold the MAPE value is computed. The average of the MAPE’s *k* values across the k-folds for a given architecture represents the performance of that architecture. The architecture with the lowest average is the best. It represents the model that has to be trained on all data. The *k-fold cross-validation method* works as described by the following pseudo-code:

**START:**

#split data into training and testing dataset

trainData, testData = split(allData)

#tune parameters of the model

parameters = …

k = …

archSkills = list()

**for** p in parameters **do**

 k-fold_skills = list()

 **for** i in **do**

   k-fold _train, k-fold_val = split(i, k, trainData)

   model = fit (k-fold-train, p)

   skill_estimate = evaluate(model, k-fold_val)

 **end for**

 *# for each k-fold calculate MAPE and store the average*

 skill = summarize(k-fold_skills)

 archSkills.append(skill)

**end for**

#evaluate the model with the best tuning with all data

model = fit(trainData)

skill = evaluate(model, testData)

**END**.

**Figure 5:**
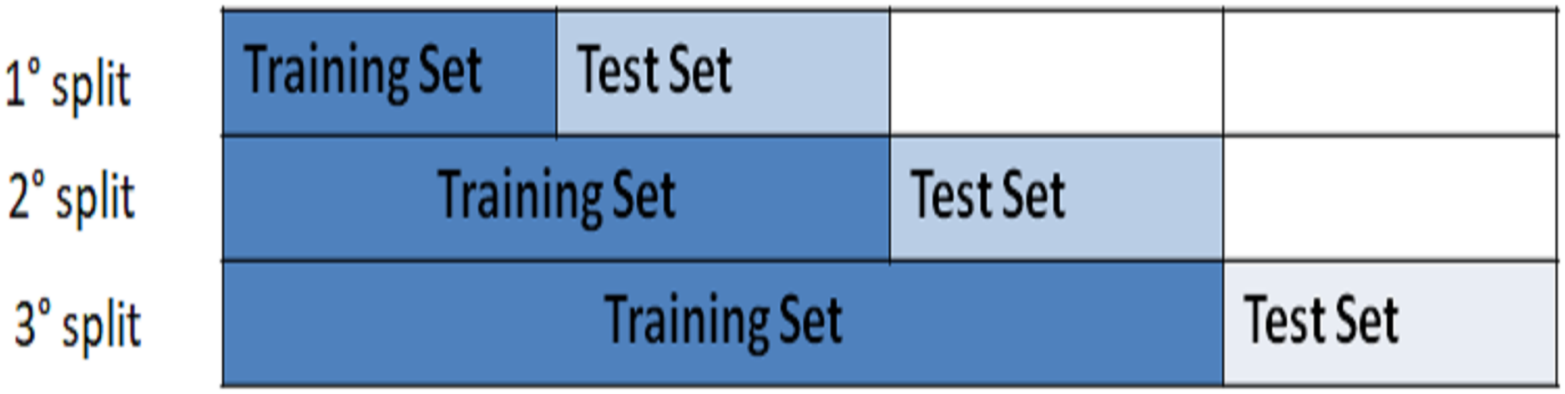
The figure shows as 70% of the whole data set is divided into 3-folds/splits, and as the expanding window approach, used to cross validation, works.

We applied the *k-fold cross-validation method* with *k* = 3–70% of the whole dataset. We used the k-fold method with an expanding window, hence we divided the data set in three folds/splits as illustrated in Fig. 5^8^ 8We tested the k-fold method with both an expanding window and a sliding window, and at the end we chose an expanding window given that with it we obtained the best results.. As underlined by Kuhn & Johnson (2013) a formal rule to choose the value of *k* does not exist. Since our data set size is not large enough *three* should be an acceptable value for *k*. We repeated the *k-fold cross-validation method* several times, choosing at the end the tuning that provides the architecture having the best performance.

As regards the hyper parameters of the BNN, one, two, and three hidden layers, *n*_*hl*_, were tested, with combinations of 50, 100, 200, 300, 400 and 500 neurons, *n*_*n*_. As regards the hyper parameters of the other ML techniques adopted (FFNN, LSTMNN and SVR), they were selected running sixty Monte Carlo runs, and taking in each run a different constellation. The hyper parameters that must be selected, are the number of epochs, *n*_*e*_, the number of neurons, *n*_*n*_, the number of batches, *n*_*b*_, the degree of the polynomial kernel, *d*, the value of *γ* (that is a parameter of the radial basis kernel). For each of them we defined the range in which they vary, as follows^9^ 9Note that all the parameters not mentioned here are defined as described in Section *Proposed Frameworks* or, given that we used the well known python libraries, *sklearn* and *keras*, are set equal to the default values.: *n*_*e*_ ∈ [50, 500], *n*_*n*_ ∈ [20, 100], *n*_*b*_ ∈ [5, 100], *d* ∈ [1, 10], γ ∈ [0.1, 100].

Once the best architecture is selected, we train it with all data, considering the testing set equal to 30% of the whole data set, that is the part of the data set not used in the k-fold method. To evaluate the robustness of the selected architecture’s performance we repeated this procedure forty times, applying the so called Monte Carlo method. The performance of each proposed framework are measured by the average and the standard deviation of the MAPE’s values across the Monte Carlo runs^10^ 10It is worth to underline that a classification model could also be considered, since low MAPE values do not necessarily mean that the model predicts the price rise and fall correctly. Classification models will be taken into account in our future work, but they are out of scope of this first paper.^,^^11^ 11In this work we trained all our frameworks for some days on a laptop with an Intel(R) Core(TM) i5-7200U CPU @ 2.50 GHz 2.71 GHz, 16 GB RAM and Graphic card Intel HD 620..

## Results

As just described, to tune our frameworks we applied the *k-fold cross-validation method* to 70% of the whole data set, using *k* = 3, an expanding window, and respecting the temporal order of the series. For each defined architecture, we applied this method computing the prediction at (*t* + *n*)th day ahead of time, with *n* = 1 and the MAPE’s values for each fold.

The *k-fold cross-validation method* was run once for each different constellation of the main parameters of each framework, since our goal is to select the best model/architecture for ANN and the best model for BNN, in order to compare the performance of these two kind of NNs.

Table 2 reports the measure of the best performance for each proposed framework, and their hyper parameters, *n*_*e*_, *n*_*n*_, *n*_*b*_, *γ*, *d*, and *n*_*hl*_. Precisely for all frameworks under study, the average and the standard deviation (values in brackets) of the MAPE’s values across the k-folds are described.

**Table 2:** Parameters and statistics of the best selected architecture for each proposed framework, in order to predict the Bitcoin price, are described. Statistics represent the average and standard deviation (in brackets), across the k-folds with *k* = 3, of the MAPE values. Note that (r) stands for SVR with a radial kernel function, (l) stands for SVR with a linear kernel function, and (p) stands for SVR with a polynomial kernel function. The bold entries highlight the framework with the lowest average of the MAPE’s values across the k-folds.

	LSTMNN	SVR (r) + LSTMNN	SVR (l) + LSTMNN	SVR (p) + LSTMNN
# *epochs*	83	330	319	136
# *neurons*	50	22	99	93
# *batchs*	40	96	87	98
γ		11.53		
*d*				1
avg (std)	5.79 (1.00)	6.76 (1.56)	**2.66 (0.08)**	2.7 (0.31)

	FFNN	SVR (r) + FFNN	SVR (l) + FFNN	SVR (p) + FFNN
# *epochs*	224	440	421	490
# *neurons*	30	68	98	85
# *batchs*	43	17	98	47
γ		0.17		
*d*				1
avg (std)	5.72 (1.39)	3.25 (0.06)	2.96 (0.51)	3.44 (0.65)

	BNN	SVR (r) + BNN	SVR (l) + BNN	SVR (p) + BNN
*n*_*hl*_	2	2	2	2
# *neurons*	300	300	300	300
γ		55.45		
*d*				3
avg (std)	**7.7 (0.39)**	9.46 (4.34)	8.49 (3.34)	207 (171.83)

The analysis performed highlighted the following patterns (see Table 2). Firstly, the two stages frameworks perform better than the correspondent one stage frameworks, but for the BNN and SVR(r)+LSTMNN. Secondly, the ANN’s performance is higher than those of BNN. Thirdly, the two stages frameworks composed of SVR, using a linear kernel function performs better than other two kinds of kernels.

For ANN the best architecture selected corresponds to a two stages framework, composed of SVR + LSTMNN, in which the SVR uses a linear kernel function and the LSTMNN uses for training *n*_*e*_ = 319, *n*_*n*_ = 99 , and *n*_*b*_ = 87. For this framework we obtained the lowest average of the MAPE’s values across the k-folds. This average value is equal to 2.66 (*std*. 0.08).

Contrary to ANN, for BNN the *k-fold cross-validation method* identifies, as the best architecture, the one stage framework, in which the BNN follows the configuration described in section *Bayesian Neural Network*, with two hidden layers and 300 neurons. For this framework the average of the MAPE’s values across the k-folds, is equal to 7.7 (*std*. 0.39).

Once selected the best models we trained the model using the whole data set and compared the results. In order to evaluate the robustness of the selected models we run forty Monte Carlo runs, computing for each run the MAPE value at (*t* + *n*)th day ahead of time, with *n* = 1, *n* = 10, and *n* = 20.

For ANN, and specifically, for the SVR+LSTMNN the average and standard deviation of the MAPE’ values across Monte Carlo simulation are equal to 1.95 and 0.18, to 5.94 and 0.04, and to 6.33 and 0.13, respectively for *n* = 1, *n* = 10, and *n* = 20 day ahead of time.

For BNN the average and standard deviation of the MAPE’ values across Monte Carlo simulation are equal to 1.74 and 0.09, to 3.85 and 0.24, and to 10.2 and 0.4, respectively for *n* = 1, *n* = 10, and *n* = 20 day ahead of time. Of course all these values of MAPE increase while *n* goes from 1 to 10 and to 20, since the performance decreases while the day ahead of time increases (see Table 3)^12^ 12Note that the procedure for tuning the model hyperparameters using the k-fold cross-validation method was applied considering the BTC price prediction at (*t* + *n*)th day ahead of time, with *n* = 1. It has to apply for each *n* ! = 1 to select for each *n* the best architecture..

**Table 3:** Average and standard deviation (in brackets) for MAPE values across the performed MC runs obtained by training the selected best architectures using the whole data set to predict Bitcoin price.

	SVR (l) + LSTMNN	BNN
*n* = 1	1.95 (0.18)	1.74 (0.09)
*n* = 10	5.94 (0.04)	3.85 (0.24)
*n* = 20	6.33 (0.13)	10.2 (0.4)

These results show that predicted bitcoin prices by the BNN with *n* = 1 have a MAPE very similar to that found by Mallqui & Fernandes (2019), albeit the periods analyzed are different. In fact, our work considers time series that range from January 1st, 2017 to April 31st, 2020; Mallqui & Fernandes (2019) considered two different periods. They found a MAPE value equal to 1.91 for the first interval, ranging from August 19th, 2013 to July 19th, 2016, and equal to 1.81 for the second interval ranging from April 1st, 2013 to April 1st, 2017, very close to our MAPE value of 1.74 and 1.95 respectively obtained applying BNN and SVR+LSTM. Our predicted BTC prices are also very close to that finding in the work by Mudassir et al. (2020) by applying the so called SVM and considering the interval from April 1, 2013 to December 31, 2019, hence considering also the BTC prices after April 2017, as we did. The highest volatility of the BTC prices was after April 2017, as underlined in the work just quoted. Mudassir et al. (2020) considered three data intervals: from April 1, 2013 to July 19, 2016; from April 1, 2013 to April 1, 2017; and the interval from April 1, 2013 to December 31, 2019. For the last of the three intervals the authors found a MAPE value of 3.78 for ANN, of 3.61 for LSTMNN, 1.44 for SVM, and of 2.73 for SANN. Lower MAPE values were found for the first two intervals. This is because the BTC prices volatility is not much high, and BTC prices are relatively stable, even if in the second interval BTC prices are noticeably higher toward the end.

Similar analysis was performed also for another cryptocurrency, specifically for Ethereum. Obtained results are shown in Tables 4 and 5.

**Table 4:** Parameters and statistics of the best selected architecture for each proposed framework, in order to predict the Ethereum price, are described. Statistics represent the average and standard deviation (in brackets), across the k-folds with *k* = 3, of the MAPE values. Note that (r) stands for SVR with a radial kernel function, (l) stands for SVR with a linear kernel function, and (p) stands for SVR with a polynomial kernel function. The bold entries highlight the framework with the lowest average of the MAPE’s values across the k-folds.

	LSTMNN	SVR (r) + LSTMNN	SVR (l) + LSTMNN	SVR (p) + LSTMNN
# *epochs*	212	172	445	199
# *neurons*	100	83	44	35
# *batchs*	21	20	54	21
*γ*		66.78		
*d*				1
avg (std)	6.04 (1.6)	6.44 (2.18)	3.44 (0.61)	3.6 (0.58)

	FFNN	SVR (r) + FFNN	SVR (l) + FFNN	SVR (p) + FFNN
# *epochs*	85	295	284	284
# *neurons*	95	31	99	99
# *batchs*	19	59	56	56
*γ*		76.56		
*d*				1
avg (std)	5.73 (1.12)	7.53 (2.89)	3.31 (0.54)	**3.26 (0.53)**

	BNN	SVR (r) + BNN	SVR (l) + BNN	SVR (p) + BNN
*n*_*hl*_	2	2	2	2
# *neurons*	300	300	300	300
*γ*		49.31		
*d*				3
avg (std)	**4.91 (2.7)**	13.2 (8.21)	8.1 (3.43)	54.1 (46.6)

**Table 5:** Average and standard deviation (in brackets) for MAPE values across the performed MC runs obtained by training the selected best architectures using the whole data set to predict Ethereum price.

	SVR (p) + LSTMNN	BNN
*n* = 1	2.84 (0.76)	2.77 (0.61)
*n* = 10	8.45 (1.00)	4.03 (0.15)
*n* = 20	8.45 (1.78)	8.87 (0.68)

Results described in Table 4 highlight that the two stages frameworks for ANN, composed of SVR, using linear and polynomial kernel functions, perform better than the correspondent one stage frameworks and two stages framework composed of SVR, using a radial kernel function; and the BNN’s performance is higher than all the others.

Table 5 describes the average and standard deviation of the MAPE’s values across Monte Carlo simulations for the best selected architecture, SVR+FFNN and BNN. Best results are obtained for BNN as in the case of Bitcoin even if the MAPE value is slightly higher for Etherem, 2.77 (0.61) vs. 1.74 (0.09) for Bitcoin. Note that also in this case MAPE values increase while *n* goes from 1 to 20.

We conclude this section presenting the predicted Bitcoin prices at (*t* + 1)th day ahead of time by the BNN in one of the Monte Carlo simulations performed. Table 6 describes the mean, the standard deviation, and the 0.01 and 0.99 quantiles of just ten predicted values, and gives an idea of how much the predicted values differ from the true values for *n* = 1. The first column in the table describes the actual value of the Bitcoin price. Specifically these prices refer to the bitcoin price from May 12th, 2019 to May 21st, 2019. They are the first ten values of the Bitcoin price into the testing dataset. The second column describes the average values predicted by the BNN for each actual value reported in the first column. Remember that the output of the BNN are assumed to be sampled from a distribution, consequently to give an idea of the results’s goodness in the Table we illustrate for each predicted price also the standard deviation, and the 0.01 and 0.99 quantiles. For this Monte Carlo simulation the MAPE value is equal to 1.71.

**Table 6:** Statistics on ten samples of predicted bitcoin price expressed in US$ at (*t* +*n*)th day ahead of time, with *n* = 1, using BNN. Note that these values refer to the first ten samples of the testing dataset. The corresponding actual values are shown in the first column.

Actual value	Mean	Std	0.01 Quantile	0.99 Quantile
7,169.8	7,162	50.5	7,038.9	7,275.1
7,646.2	7,586.8	56.5	7,473.7	7,716.9
7,990.4	7,860.1	58.4	7,715.1	7,992.8
8,062.6	7,913	56.6	7,787.6	8,045.3
7,983.7	7,688.4	46.6	7,600.1	7,801.6
7,196.2	7,600.1	44.9	7,500.3	7,713.1
7,328.6	7,723.7	48.4	7,610.6	7,843.6
7,943.2	7,835.7	46.5	7,733.1	7,936.3
7,902.4	7,916.7	46.4	7,814.6	8,047.5
7,950.6	7,876.6	47.6	7,772.9	8,001.2

## Conclusions

In this article, several ML frameworks to forecast Bitcoin and Ethereum prices are comparatively tested. They are divided into one stage frameworks and two stages frameworks. The formers are frameworks based on just one ML technique. The latters are based on two ML techniques working in cascade. We used three one stage frameworks, and three two stages ones. The first three use different NN models. Specifically, we considered BNN, FFNN and LSTMNN. The second ones use an FFNN, an LSTMNN, and an BNN, each of them in cascade to an SVR.

The goal of this work was to analyze the performance of BNN in the forecasting the Bitcoin and Ethereum daily closing prices, and to compare it with those obtained using FFNN and LSTMNN, considering both the typologies of frameworks. All frameworks attempt to predict the prices starting from five technical indicators, SMA, EMA, MOM, MACD, and RSI.

Specifically, in the one stage frameworks starting from the value of these five technical indicators at *t*th day, we predicted the daily closing price at (*t* + *n*)th day, with *n* = 1, *n* = 10, and *n* = 20. In the two stages frameworks the first stage, formed by an SVR, receives in input the five technical indicators at *t*th day and predicts the value of the five technical indicators at (*t* + *n*)th day; the second stage receives in input the estimate of five technical indicators at (*t* + *n*)th day and predicts the daily closing price at (*t* + *n*)th day.

We tuned all the proposed framework applying the *k-fold cross-validation method* to 70% of the whole data set. We created several models training them at each k-fold, hence computing for each fold a MAPE’s value. Then, for each model we computed the average of the MAPE’s values across the k-folds. The model with the lowest average results to be the best. It represents the model that has to be trained on all data. We selected the best model for the ANN and the best model for BNN. The former corresponds to a two stages framework, and the latter corresponds to a one stage framework, both for Bitcoin and Ethereum price prediction.

Results show that the predicted bitcoin prices by the BNN have a MAPE in accord with those reported in the works present in the literature. Furthermore, the performance of some proposed two stages frameworks, *SVR*+*FFNN* and *SVR*+*LSTMNN*, show a clear improvement with respect to those of the correspondent one stage frameworks, and the goodness of some two stages frameworks’ predictions is close to that obtained by the BNN.

The goal of this work is to give useful insights to build efficient frameworks for trading. The proposed frameworks could be used to decide when and how much to invest, and also to build efficient bitcoin trading strategies in a market highly volatile, in which short term trading may give several opportunities to make profit when correct trading strategies are applied.

The novelty of this work consists in the model selection, by applying the *k-fold cross-validation method* to 70% of the whole dataset, and in applying the Monte Carlo method during the training phase of the best selected architectures that takes the whole dataset into account, to predict cryptocurrency markets, specifically the Bitcoin and the Ethereum market.

Future work aims to perform a more exhaustive optimization of all the proposed frameworks in this work, and to analyze their response in the case in which more inputs are taken into account. In fact, it is reasonable thinking that a more exhaustive optimization of the proposed frameworks and more inputs to train the networks will allow to obtain even higher performance.

## Supplemental Information

10.7717/peerj-cs.413/supp-1 Supplemental Information 1 Files containing the simulations' results and time series used. File named README describes the files uploaded.

## Additional Information and Declarations

### Data Availability

The following information was supplied regarding data availability: Data files are available in the Supplemental Files.
