# Supplementary material for: Predictions of bitcoin prices through machine learning based frameworks
Source: PeerJ Comput Sci. 2021 Mar 29;7:e413. doi: 10.7717/peerj-cs.413 (PMC8022579; doi:10.7717/peerj-cs.413)
Supplement: Supplemental Information 1 — File named README describes the files uploaded. [file peerj-cs-07-413-s001.zip › RESULTS/README.pdf]

The uploaded files are divided in two directories named “Ethereum” and “Bitcoin containing similar files. Ethereum directory contains files related to Ethereum price time series, and Bitcoin directory contains files related to Bitcoin price time series. In turn each of these two directories is divided in “BNN_files” and “ANN_files”.
The directory named “BNN_files” contains the data related to the predicted bitcoin prices at (t + 1) -th day ahead of time by the BNN in one of the Monte Carlo simulations performed. Precisely, it contains the file named “predicted price avg” that illustrates the mean, the file “predicted price std” that illustrates the standard deviation, and the “0.01 quantiles” and “0.99 quantiles” files that illustrate respectively the 0.01 and the 0.99 quantiles of the predicted bitcoin price.
The directory named “ANN_files” contains the results data about the 60 runs related to the k-fold cross-validation method. These results are illustrated in the files named “ANN_k_fold_3_day_1”, and “iperparameters_k_fold_3_day_1”.
The files “MAPE AVG and STD for LSTM and SVR(rbf,lin,poly)+LSTMNN” and “MAPE AVG and STD for LSTM and SVR(rbf,lin,poly)+LSTMNN” describe the performed analysis on the results in the first file above mentioned that is in the file named “ANN_k_fold_3_day_1”.
The three files named “SVR(lin)+LSTM_day_1”, “SVR(lin)+LSTM_day_10” and “SVR(lin)+LSTM_day_20” describe the results of the 40 Monte Carlo runs, performed to evaluate the robustness of the model selected through the k-fold cross-validation method.

The remaining two files named “prices” and “statistics” describe the price time series and the summary statistics of the inputs of our proposed frameworks, respectively.
